# The Actual Cautery

**Published:** 1901-03-16

**Authors:** 


### THE ACTUAL CAUTERY.

Everyone likes to have a reason for what he does, and it is possible that much of the modern neglect of counter irritation is due to the obscurity which overhangs its mode of action. In an address lately delivered before the Hunterian Society, Sir William M. Banks twitted the profession on running after new things while old and well tried remedies were left unused, and insisted on the utility of the actual cautery. The cases which he mentioned as illustrating the surprising efficacy of the cautery in the treatment of chronic inflammations, and more especially in relieving the pains by which they are sometimes accompanied, included syphilitic periostitis, various inflammatory joint diseases, more particularly those of traumatic origin, and spinal inflammation after injury.

But the subject to which he specially devoted his attention was the utility of the actual cautery in the treatment of pruritus ani. Pruritus ani may, he says, be set up by various causes, which, however, in his experience, have practically been narrowed down to diabetes, to eczema, to irritation mainly due to spirit drinking, and to a condition which may be described as "idiopathic—pathology unknown." The first are capable of being removed and often cured by appropriate constitutional treatment; but the last cause, which is the one associated with the worst cases, is unfortunately quite inexplicable as yet. Nothing special can be found in the skin of the part affected. There may be small skin piles present, but they are never much to look at. Fissure or fistula there is none, nor internal piles either. The appearances most commonly noticed are that the perineal skin is thrown into fine folds which are hypertrophied states of the natural tendency to wrinkling round the orifice. The commencement of the perineal raphe shares in this. There is no particular amount of wetness to be seen, but it is noticeable that the affected area has the appearance of being made of slightly damp rugose washleather. Certainly the condition does not seem to have anything to do with want of cleanliness, the patients in whom it was met with being all persons of remarkably cleanly habits, and belonging to the better ranks in life. In regard to treatment there is a certain limited number of cases in which no drugging or dieting seems to have the least effect. In these cases the torture of the itching becomes most exquisite, so that the patient's health may be seriously impaired from worry and want of sleep; and for these "idiopathic" cases the actual cautery affords a prospect of cure when everything else has failed. Thoroughness of application is essential. The patient should be chloroformed and tucked up in the lithotomy position. Any small skin piles should be clipped away with the scissors. If there are not many rugæ, then the large bulbous-headed cautery, which is usually found in Paquelin's case, should be used, and the skin for an inch and a half all round the anal orifice well frizzled. If there are many rugæ then the small cautery point should be used, and it should be introduced into the furrows between the rugæ as well as applied over the tops of them.

In one of the cases referred to, the rugæ were so pronounced that the tops of them were cut off with a pair of curved scissors. After describing various cases in which this treatment had been remarkably successful, Sir William M. Banks said: "It is only in very severe cases for which no reasonable cause can be found that I venture to recommend the cautery. It is about twenty years or more since I tried the remedy for the first time, and during all the years that have intervened I do not think that I have used it more than a dozen times altogether. But the very value of the remedy lies in its being unfailingly curative in absolutely intractable cases."

